# What is the impact of endometriosis and the AFS stage on cumulative pregnancy rates in IVF programs?

**DOI:** 10.1186/s12978-024-01747-8

**Published:** 2024-01-29

**Authors:** Karine Morcel, Philippe Merviel, Sarah Bouée, Mathilde Le Guillou, Marine Carlier, Pandora James, Hortense Drapier, Damien Beauvillard

**Affiliations:** 1 Reproductive Medicine Department, ART Center, Brest University Medical Center, 2 Avenue Foch, 29200 Brest, France; 2 Reproductive Laboratory Department, ART Center, Brest University Medical Center, 2 Avenue Foch, 29200 Brest, France

**Keywords:** Endometriosis, AFS stage, Endometrioma, IVF, Pregnancy rate

## Abstract

**Background:**

Endometriosis is commonly observed in infertile women and can be staged with regard to severity [e.g. according to the American Fertility Society (AFS) classification]. This condition can cause infertility through impaired oocyte quality, fertilization disorders, tubal lesions, adhesions, deep infiltration, and adenomyosis. Although women with endometriosis often turn to in vitro fertilization (IVF) programs, the literature data on IVF outcomes are sometimes contradictory (i.e. the same as in other etiologies of infertility, or worse). The objective of the present study was to assess and compare pregnancy rates in women with and without endometriosis and according to the endometriosis stage.

**Methods:**

We retrospectively studied clinical and ongoing pregnancy rates in IVF and the cumulative pregnancy rates after frozen/thawed embryo transfers, in women without endometriosis (group A) or with endometriosis (group B). We further compared groups in which endometriosis was staged according to the revised AFS classification: stage 1/2 (group C), stage 3/4 (group D, without endometrioma), and endometrioma alone (group E).

**Results:**

We documented 430 cycles in group A and 460 in group B (including 56 in group C, 88 in group D and 316 in group E). After fresh or frozen/thawed embryo transfers, the differences in ongoing pregnancy rates between groups A and B were not significant. However the cumulative rates per couple were significantly lower (p < 0.05) in group D.

**Conclusions:**

We recommend IVF for women with endometriosis because the pregnancy rates are similar to those observed for women with other types of infertility. This approach is in line with the international guidelines issued by assisted reproductive technology societies. These results again raise the question of whether surgical resection of deep infiltrating endometriosis (stage 3/4) should be recommended before admission to an IVF program.

*Trial registration* This study was approved by an institutional review board (CPP Ouest VI, Brest, France): reference: B2020CE.43

## Background

Endometriosis is a pathology characterized by the presence of endometrial tissue outside the uterus. Since Sampson published his theory (the menstrual dissemination of endometrial tissue into the peritoneal cavity) in 1927 [1], the pathophysiology of endometriosis has been studied extensively with regard to the influence of genetic, epigenetic, hormonal, inflammatory and environmental factors. The estimated prevalence of endometriosis is 10–15% in the general female population and as much as 50% among infertile women [2]. Cases of endometriosis can be classified with regard to severity [3]: minimal/mild endometriosis (stages 1 and 2 in the American Fertility Society (AFS) classification) cause infertility through abnormalities in oocyte quality, fertilization disorders, and the secretion of intrapelvic cytokines. In this case, a meta-analysis showed that the resection of endometriotic lesions is associated with improved natural fertility [4]. In AFS stages 3 and 4 (moderate and severe endometriosis), infertility may result from intrapelvic adhesions, tubal lesions, ovarian cysts (endometriomas), deep infiltrating endometriosis (DIE), and uterine abnormalities (adenomyosis). The value of surgical treatment of these lesions is subject to debate. Other disturbances that might affect fertility include implantation failure, progesterone resistance, and early pregnancy loss [5]. All these abnormalities can influence the pregnancy rates in in vitro fertilization (IVF).

Although over 70% of women with endometriosis will become pregnant spontaneously, there are international guidelines on managing infertility in a setting of endometriosis [6]. Depending on the endometriosis stage, one can consider either three to six intra-uterine inseminations or an immediate switch to IVF [and, if necessary, intracytoplasmic sperm injection (ICSI)]. IVF is necessary in 10 to 25% of women with endometriosis, and the outcomes vary from one study to another. In 2002, Barnhart reported particularly poor outcomes for women with endometriosis (twice as bad as for women with other indications for IVF) [7], while an analysis of the Society for Assisted Reproductive Technology registry [8] (covering 450,000 IVF cycles between 2004 and 2008) showed that the IVF outcomes for women with endometriosis were similar to those observed for couples with other causes of infertility. Other researchers have shown that stage 3/4 endometriosis has a negative impact [9–11] or no impact [12, 13] on the outcome of IVF.

In view of these disparities, the objective of the present study was to compare clinical and ongoing pregnancy rates in women with vs. without endometriosis, as a function of the endometriosis stage (1/2 vs. 3/4 vs. endometrioma). Endometriomas were classified usually as stage 4 endometriosis. Another of our study’s objectives was to establish whether endometrioma alone was associated with a higher pregnancy rate. Lastly, we studied the cumulative pregnancy rates after frozen/thawed embryo transfers.

## Methods

### Study design

Between January 1st, 2019, and October 31st, 2022, we included women aged 18 to 42 (with or without pelvic endometriosis) participating in an IVF program, and sometimes with frozen/thawed embryo transfer at our assisted reproductive technology (ART) center. The diagnosis of endometriosis and the AFS/American Society for Reproductive Medicine (ASRM) staging were based on the clinical signs and symptoms and confirmed by MRI and/or laparoscopy. Stage 1/2 endometriosis was diagnosed during a laparoscopic assessment, and the lesions were then resected and/or treated with electrocoagulation. A diagnosis of stage 3/4 endometriosis was based on MRI or laparoscopic findings. In some cases, the lesions were resected during the laparoscopy. Ovarian endometriosis (with single or bilateral endometrioma) was diagnosed by MRI and (when the cyst was greater than 60 mm in diameter) treated with intraperitoneal kystectomy. We excluded (i) women with uterine cavity abnormalities or adenomyosis and (ii) couples in gamete or embryo donation programs. During the same period, we selected endometriosis-free women consulting at our ART center and who were matched with the women with endometriosis by age, body mass index (BMI), smoking status, and primary or secondary infertility. Thus, we divided the study population into five groups: women without endometriosis (group A), women with endometriosis (all stages; group B), women with stage 1/2 endometriosis (group C), women with stage 3/4 endometriosis (without endometrioma; group D), and women with endometrioma alone (group E).

All couples enrolled in the IVF program cycles underwent the same infertility assessment. For women, this included hormone blood tests on the second or third day of the menstrual cycle (including assays of serum follicle stimulating hormone (FSH), luteinizing hormone (LH), estradiol (E2), prolactin, and anti-Müllerian hormone (AMH) levels) and hysterosalpingography (followed by hysteroscopy if the uterine cavity was abnormal). The most recent AMH level was recorded, unless it had been measured more than 3 months after surgery. For men, a sperm motility test was required. All the hormone blood tests and sperm examinations were performed in the same laboratory. Groups A to E were then compared with regard to the demographic and clinical data, ovarian stimulation and pregnancy outcomes.

### Stimulation protocols

#### The IVF cycle

Three different controlled ovarian stimulation protocols (depending on the woman’s profile and the physician’s clinical preferences) were prescribed during the study period. In the long gonadotropin-releasing hormone (GnRH) agonist protocol, triptorelin (Decapeptyl®, Ipsen Pharma, Paris, France) was administered on day 20 of the previous cycle at a dose of 0.1 mg per day and then (starting 14 days later) 0.05 mg per day, with FSH or human menopausal gonadotropin (hMG) stimulation. In the short GnRH agonist protocol, triptorelin was administered on day 2 of the stimulated cycle (0.1 mg per day), with FSH or hMG stimulation. In the GnRH antagonist protocol, stimulation with FSH or hMG started on day 2 of the cycle, with the initiation of 0.25 mg per day ganirelix (Orgalutran®, MSD, Levallois-Perret, France) when the follicle size exceeded 14 mm or when the estradiol (E2) level was over 400 pg/mL. The dose of gonadotropin (FSH or hMG) was adjusted after the first evaluation on day 5 (in antagonist protocols) or day 7 (in agonist protocols) of gonadotropin administration. The evaluation combined hormone assays (estradiol-17β, LH, and progesterone) and vaginal ultrasound assessment (the number and size of follicles and the endometrial thickness and maturation). This evaluation was repeated every 2 or 3 days, depending on the follicular growth. When (i) at least three follicles had grown to a diameter of 17 mm or more, (ii) the endometrium was 7 mm or more thick, and (iii) a triple-line pattern was observed, a dose of 250 µg of recombinant hCG (Ovitrelle®, Merck, Lyon, France) was administered. Oocytes were retrieved 35 h after hCG administration. Luteal phase supplementation consisted of 400 mg per day intravaginal micronized progesterone (Utrogestan®, Besins International, Paris, France) from the evening of oocyte retrieval until the day of the β-hCG assay 2 weeks later.

Adequate, cleaved embryos were defined as those with both normal-sized, normally shaped blastomeres, and a fragmentation rate of 10% or less [14]. Blastocysts were evaluated according to Gardner’s classification [15]. The embryos were transferred in utero on day 2, 3 or 5, using a Frydman catheter (CCD Laboratories, Paris, France). The oocyte or embryos were all frozen in cases with (i) an elevated (> 1.5 ng/mL) plasma progesterone level on the hCG trigger day, (ii) ovarian hyperstimulation (> 3500 pg/mL and/or > 20 oocytes), or (iii) a lack of spermatozoids on the retrieval day.

Other good-quality embryos were frozen by vitrification, using the Vitrification Freeze Kit® [dimethyl sulfoxide, ethylene glycol, sucrose; Fujifilm Irvine Scientific (Santa Ana, CA USA)] from 2019 to January 2022 and thereafter with the Vitrification Freeze Kit-NX® (dimethyl sulfoxide, ethylene glycol, trehalose; Fujifilm Irvine Scientific). All early day 2/day 3 embryos were packaged in closed-system straws (VHS®, CryoBioSystem, L'Aigle, France), whereas blastocysts (day 5) were frozen in open system vitrification carriers (VitrifitTM, CooperSurgical (Ballerup, Denmark) from December 2021 onwards. Typically, the embryos were immersed for 10 min in the equilibrium solution and for 30 s in the freezing solution before being packaged and immersed in nitrogen. Regardless of the packaging system or vitrification kit, all straws were thawed with a Thaw Vitrification kit® (sucrose; Fujifilm Irvine Scientific). The embryos were immediately thawed and placed for 1 min in the thawing solution, for 4 min in a dilution solution, and then for 2 × 4 min in a rinsing solution before being grown in culture medium (SAGE 1-Step®, CooperSurgical) supplemented with 12 mg/mL human serum albumin (SAGE-HSA®, CooperSurgical) for no more than 4 h.

#### The frozen-thawed embryo transfer cycle

Two frozen-thawed embryo transfer protocols were implemented during the study period. The artificial cycle protocol consisted of the administration of 6 mg/day estrogen for 12 days (Provames®, Merus Labs Luxco II, Luxembourg) and then an ultrasound measurement of the endometrial thickness; when the latter value exceeded 8 mm, 600 mg/day intravaginal micronized progesterone was added, and the embryos were transferred 2, 3 or 5 days later. The estrogen and progesterone treatments were maintained for 2 months. The stimulation cycle protocol consisted of the administration of FSH or hMG from day 2 of the cycle, with ultrasound and blood hormone monitoring on stimulation day 7. These variables were assessed every 2 or 3 days, depending on the follicles’ growth. When (i) a follicle had grown to a diameter of 17 mm or more, (ii) the endometrium was 7 mm or more thick, and (iii) a triple-line pattern was observed, a dose of 250 µg of recombinant human chorionic gonadotropin (hCG) was administered. Luteal phase supplementation consisted of 400 mg per day of intravaginal micronized progesterone from the evening of ovulation until the β-hCG assay 2 weeks later. Embryo transfer occurs 2, 3 or 5 days after ovulation.

Each clinical pregnancy was confirmed by ultrasound 6–8 weeks after embryo transfer (gestational sac and, in some cases, the embryo) and a β-hCG level above 1000 IU/L. An ongoing pregnancy was defined as pregnancy at more than 12 weeks of amenorrhea (WA). The implantation rate was the ratio between the number of gestational sacs and the number of embryo transferred. The cumulative clinical pregnancy rate (CCPR) and the cumulative ongoing pregnancy rate (COPR) per couple were calculated after all the embryos (fresh and frozen/thawed) had been transferred.

### Ethics approval and consent to participate

All couples were informed of the study and gave their written consent to participation. A copy of the signed consent was included in the woman’s medical records, and another copy was stored by the leading investigator. This study was approved by an institutional review board (CPP Ouest VI, Brest, France); reference: B2020CE.43.

### Statistical analysis

Statistical comparisons were conducted using either Student’s T-test or a Mann–Whitney U test (for continuous variables) and a chi-squared test or Fisher’s exact test (for qualitative variables). We used the XLSTAT® add-in (Addinsoft, Paris, France) for statistical analysis. The threshold for statistical significance was set to p < 0.05.

## Results

**Fig. 1:**
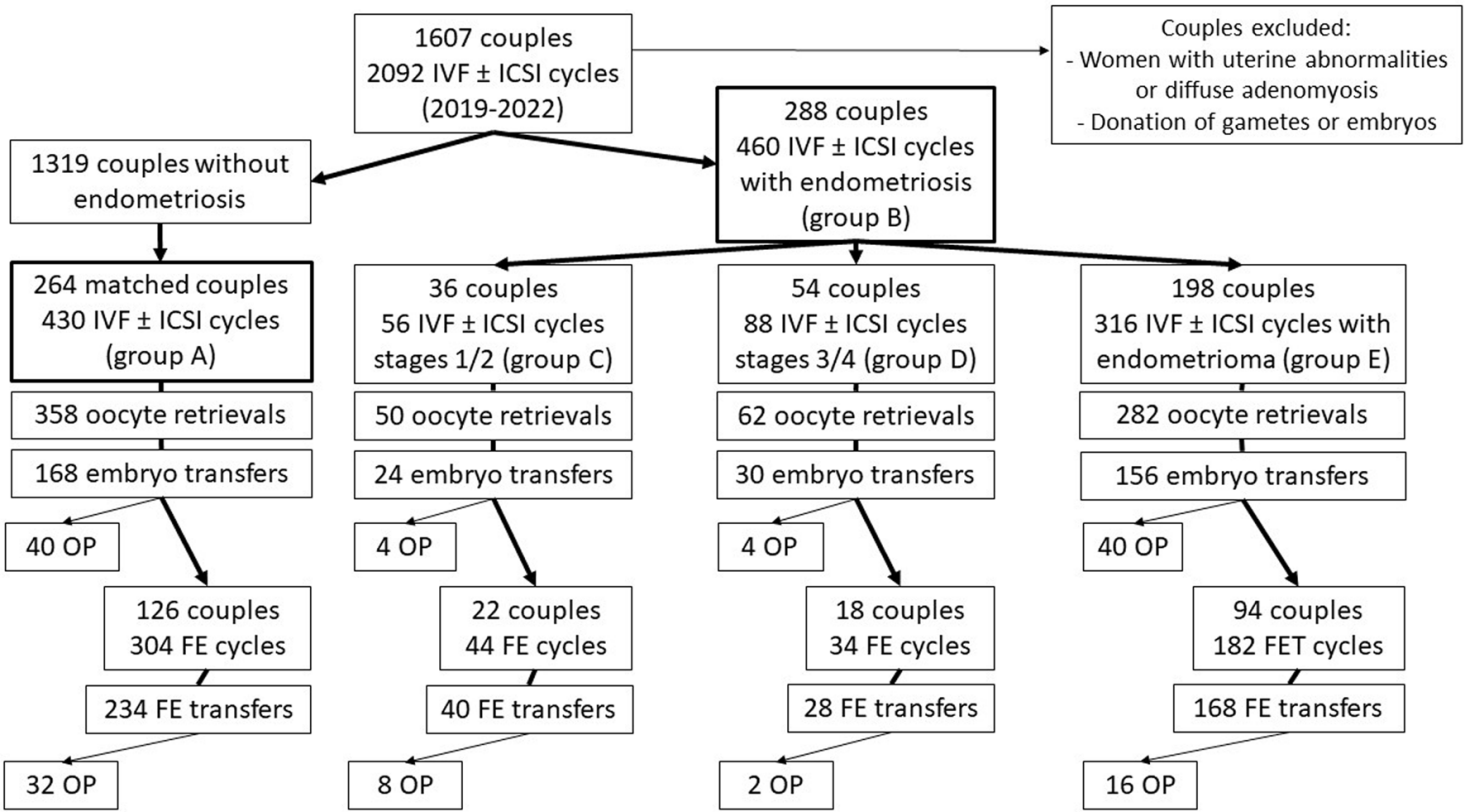
Study flow chart. Group A: women without endometriosis; group B: women with endometriosis (all stages); group C: women with stage 1/2 endometriosis; group D: women with stage 3/4 endometriosis (without endometrioma); group E: women with endometrioma alone. *OP* ongoing pregnancy, *FE* frozen embryo

Between January 1st, 2019, to October 31, 2022, we documented a total of 2092 IVF cycles in 1607 couples. We analyzed 430 cycles for the 264 couples in group A, and 460 cycles for the 288 couples in group B (Fig. 1). With regard to the endometriosis stage, we recorded 56 cycles for the 36 couples in group C, 88 cycles for the 54 couples in group D, and 316 cycles for the 198 couples in group E. After the IVF cycle, some woman received frozen/thawed embryo transfers: 304 cycles for 126 couples in group A, 260 cycles for 134 couples in group B, 44 cycles for 22 couples in group C, 34 cycles for 18 couples in group D, and 182 cycles for 94 couples in group E. During this period, a total of 1503 frozen/thawed embryo transfer cycles were performed at our ART center.

The five study groups did not differ with regard to the woman’s age, BMI, or smoking status (Table 1). The same was true for the men (data not shown). Group C differed significantly from the other groups with regard to basal levels of FSH, LH, oestradiol and AMH; this was doubtless related to the higher prevalence of polycystic ovary syndrome (PCOS) in group C. The proportion of women with Fallopian tube defects was significantly higher in group D than in the other groups. All women in group C (100%), 5 of the women in group D (9.2%), and 21 of the women in group E (10.6%; when endometrioma exceeded 60 mm) underwent laparoscopic surgery for endometriosis. In the group E, the mean size of the endometrioma was 31.2 ± 19.1 mm, and 40 women had bilateral cysts (20.2%).

**Table 1:** Demographic and clinical data

	Group A	Group B	Group C	Group D	Group E	p
N couples	264	288	36	54	198	
Woman age (years old)	34.8 ± 3.5	34.5 ± 2.9	34.2 ± 3.1	34.3 ± 3.4	34.6 ± 3.3	
Woman BMI (kg/m^2^)	23.9 ± 3.2	24.2 ± 4.7	24.4 ± 3.9	24.2 ± 4.1	24.2 ± 5.0	
Woman tobacco use (%)	19.7	22.9	28.5	20.4	22.7	
Basal FSH (IU/L)	7.4 ± 3.6	7.3 ± 2.9	6.6 ± 2.1	8.0 ± 2.7	7.3 ± 3.0	**C–D
Basal LH (IU/L)	5.3 ± 2.8	5.2 ± 2.4	6.4 ± 2.8	5.0 ± 2.3	5.1 ± 3.7	*A–C, B–C, C–D, C–E
Basal E2 (pg/mL)	40.2 ± 16.3	38.3 ± 16.8	30.1 ± 21.3	42.3 ± 30.4	38.7 ± 30.0	*A–C, B–C, C–D
Basal AMH (ng/mL)	2.1 ± 1.8	2.0 ± 1.5	2.5 ± 1.7	2.0 ± 1.3	1.9 ± 1.5	*C–E
Normal ovul (%)	81.8	81.2	72.2	77.7	83.8	
PCOS (%)	14.2	13.2	22.2	11.2	12.1	*A–C, B–C; **C–D, C–E
POR (%)	4.0	4.8	5.6	7.2	4.1	*B–D; **A–D, D–E
Normal tubes (%)	76.1	56.9	60.7	29.6	63.6	**B–D; ***A–B, A–D, C–D, D–E
Normal uterus (%)	82.5	81.6	86.1	77.7	81.8	
Primary infertility (%)	56.0	59.3	61.1	57.4	59.6	

Group A: women without endometriosis; group B: women with endometriosis (all stages); group C: women with stage 1/2 endometriosis; group D: women with stage 3/4 endometriosis (without endometrioma); group E: women with endometrioma alone

*BMI* body mass index, *PCOS* polycystic ovary syndrome, *POR* poor ovarian response

Statistical analysis: *p < 0.05; **p < 0.01; ***p < 0.001. The other comparisons were not significant

More ICSI procedures were performed in groups A and C; this was due to the lower sperm counts recorded in these groups (Table 2). In group D, the daily and cumulative doses of gonadotropin were higher but the oestradiol level on the hCG day was lower, in relation with the poor ovarian response (POR). Conversely, the oestradiol level and the endometrial thickness on the hCG day was highest in group C; again, this was related to the higher prevalence of PCOS.

**Table 2:** Ovarian stimulation data

	Group A	Group B	Group C	Group D	Group E	p
N cycles	430	460	56	88	316	
IVF and ICSI (%)	30.2/69.8	45.2/54.8	25.2/74.8	52.2/47.8	46.8/53.2	***A–B, A–D, B–C, C–D, C–E
Cycle rank (n)	1.6 ± 0.9	1.6 ± 0.9	1.6 ± 0.9	1.5 ± 0.9	1.6 ± 0.9	
Antagonist (%)	86.9	83.0	85.7	84.0	82.2	
Short agonist (%)	3.7	6.9	10.7	6.8	6.3	*B–C, C–D; **A–D, C–E; ***A–B, A–C
Long agonist (%)	9.4	10.1	3.6	9.2	11.5	***A–C, B–C, C–D, C–E
Initial FSH dose (IU/day)	272 ± 111	303 ± 123	272 ± 132	327 ± 114	303 ± 116	*C–D; ***A–B
Total FSH dose (IU)	2688 ± 1011	3059 ± 1506	2795 ± 1546	3329 ± 1344	3032 ± 1327	*C–D; ***A–B
E2 on dhCG (pg/mL)	2108 ± 1248	1916 ± 1173	2272 ± 754	1703 ± 1162	1913 ± 1229	*A–B, A–E; **A–D, B–C, C–E; ***C–D
LH on dhCG (IU/L)	3.3 ± 2.3	2.7 ± 2.1	2.0 ± 1.2	2.8 ± 2.2	2.8 ± 2.1	**A–E, C–D; ***A–B, A–C, B–C, C–E
P on dhCG (ng/mL)	0.9 ± 0.4	0.9 ± 0.4	0.8 ± 0.1	1.0 ± 0.2	0.9 ± 0.3	
Endometrium thickness (mm)	9.6 ± 2.0	9.6 ± 1.9	10.5 ± 1.7	9.7 ± 1.3	9.5 ± 2.3	**C–D; ***A–C, B–C, CE
Sperm count (×10^6^/mL)	36.4 ± 27.1	54.6 ± 42.5	36.6 ± 21.7	93.0 ± 44.0	46.3 ± 29.8	**B–E, C–E; ***A–B, A–D, B–C, B–D, C–D, D–E

Group A: women without endometriosis; group B: women with endometriosis (all stages); group C: women with stage 1/2 endometriosis; group D: women with stage 3/4 endometriosis (without endometrioma); group E: women with endometrioma alone

*dhCG* hCG trigger day

Statistical significance: *p < 0.05; **p < 0.01; ***p < 0.001. The other comparisons were not significant

The total numbers of blastocysts obtained, oocytes retrieved, mature oocytes, fertilized oocytes, embryos obtained and embryos vitrified were lower in group D than in the other groups (Table 3). The cancellation rates during ovarian stimulation and during embryo culture were respectively 16.8% and 53.1% for group A, 14.4% and 46.8% for group B, 10.8% and 52.0% for group C, 29.6% and 51.7% for group D, and 10.8 and 44.7% for group E. The proportions of “freeze-all” cases were similar in groups A and B (with 21 and 23 oocyte pick-ups, respectively) and in groups C, D and E. This was mainly due to premature progesterone elevation at the end of the ovarian stimulation (17 and 19 cases in groups A and B, respectively). The clinical and ongoing pregnancy rates after fresh embryo transfers were significantly lower in group D than in group E.

**Table 3:** Laboratory and pregnancy outcomes after fresh embryo transfers

	Group A	Group B	Group C	Group D	Group E	p
Number of oocyte pick-ups	358	394	50	62	282	
Total oocytes (n)	8.3 ± 4.4	7.9 ± 4.2	8.8 ± 4.9	7.1 ± 3.3	8.0 ± 4.9	*A–D, C–D
M2 oocytes (n)	6.9 ± 5.3	6.7 ± 5.3	7.9 ± 5.7	6.0 ± 4.1	7.1 ± 5.4	*C–D
2 pronuclei (n)	4.7 ± 3.1	4.4 ± 3.0	5.1 ± 4.3	3.7 ± 2.0	4.5 ± 2.7	*B–D, C–D; **D–E; ***A–D
Fertilization rate (%) (2 PN/M2 ooc)	68.0	65.6	64.5	61.5	63.3	
Total embryos (n)	3.9 ± 2.9	3.9 ± 3.3	4.8 ± 3.2	2.9 ± 2.4	4.1 + 3.3	**A–D, B–D; ***C–D, D–E
Cleaved/blastocyst (%/%)	40.3/59.7	39.5/60.5	32.0/68.0	51.6/48.4	38.2/61.8	***A–C, A–D, B–D, C–D, D–E
Cleavage rate (%) (emb/M2 ooc)	56.5	58.2	60.7	48.3	57.7	
Vitrified embryo (n)	2.4 ± 1.3	2.6 ± 1.4	3.1 ± 1.8	1.7 ± 0.9	2.7 ± 1.5	*A–B; **A–C, A–E; ***A–D, B–D, C–D, D–E
N fresh embryo transfers	168	210	24	30	156	
Day of fresh embryo transfers						
Day 2 or 3 (%)	17.3	16.2	8.4	23.4	7.1	***A–E, B–E, D–E
Day 5 (%)	82.7	83.8	91.6	76.6	92.9	
Embryo/transfer (n)	1.2 ± 0.4	1.3 ± 0.4	1.2 ± 0.4	1.2 ± 0.4	1.3 ± 0.4	
Clinical pregnancy	48	64	6	4	54	
EPL (n)	8	10	2	0	8	
Ectopic pregnancy (n)	0	6	0	0	6	
Twin pregnancy (n)	4	12	2	2	8	
CP/c (%)	11.1	13.9	10.7	4.5	17.0	**D–E
CP/opu (%)	13.4	16.2	12.0	6.4	19.1	*D–E
CP/transfer (%)	28.5	30.4	25.0	13.3	34.6	*D–E
IR (%)	24.7	25.6	24.1	13.8	28.5	***A–D, B–D, D–E
Ongoing pregnancy	40	48	4	4	40	
OP/c (%)	9.3	10.4	7.1	4.5	12.6	*D–E
OP/opu (%)	11.1	12.1	8.0	6.4	14.1	
OP/transfer (%)	23.8	22.8	16.6	13.3	25.6	

Group A: women without endometriosis; group B: women with endometriosis (all stages); group C: women with stage 1/2 endometriosis; group D: women with stage 3/4 endometriosis (without endometrioma); group E: women with endometrioma alone

*EPL* early pregnancy loss, *CP* clinical pregnancy, *OP* ongoing pregnancy, *c* cycle, *opu* oocytes pick-up, *t* transfer, *IR* implantation rate

Statistical analysis: *p < 0.05; **p < 0.01; ***p < 0.001. The other comparisons were not significant

**Fig. 2:**
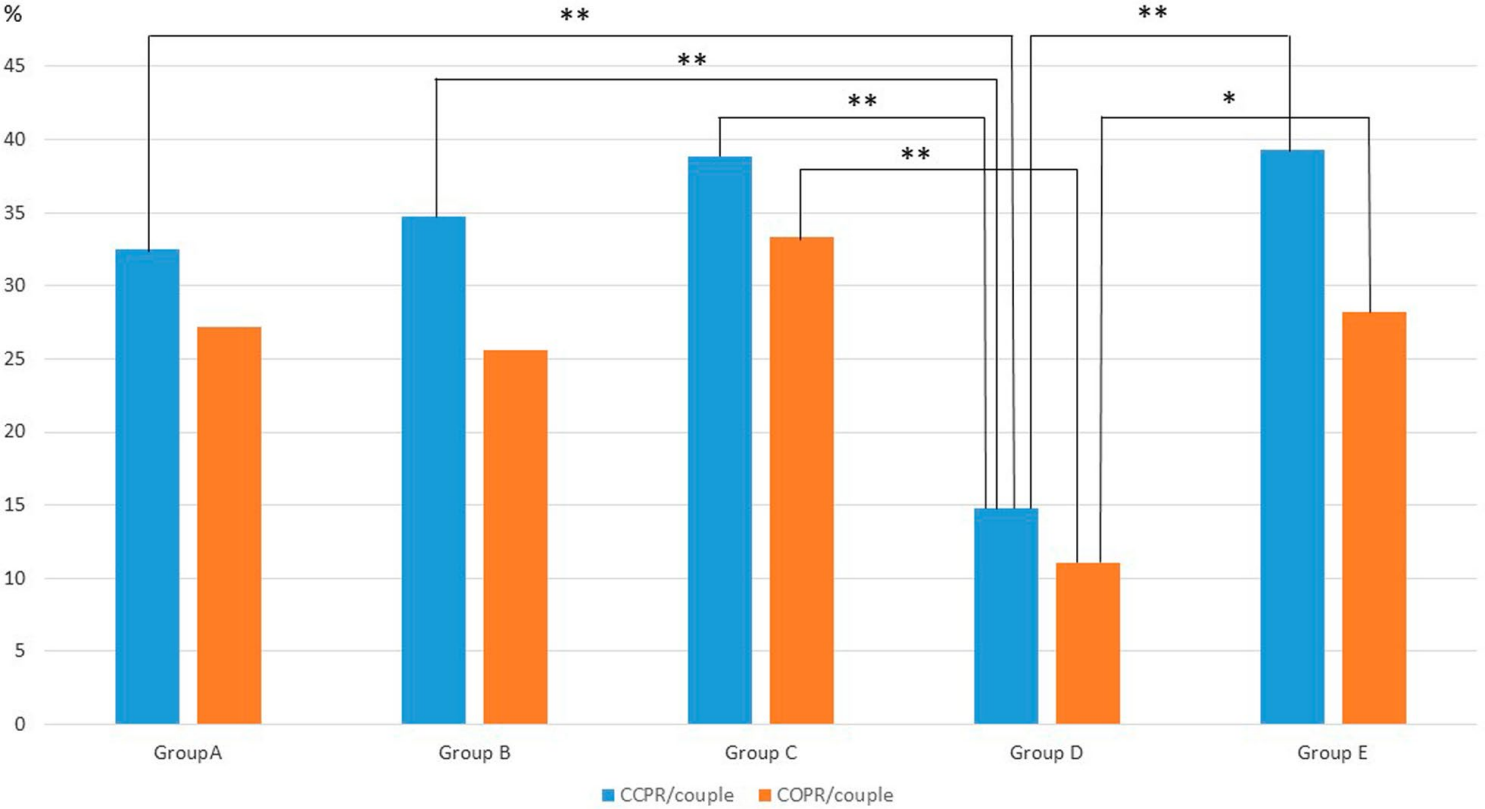
Cumulative clinical and ongoing pregnancy rates per couple with or without endometriosis and frozen embryo transfers. Group A: women without endometriosis; group B: women with endometriosis (all stages); group C: women with stage 1/2 endometriosis; group D: women with stage 3/4 endometriosis (without endometrioma); group E: women with endometrioma alone. *E* endometriosis, *CCPR* cumulative clinical pregnancy rate, *COPR* cumulative ongoing pregnancy rate. Statistical significance: *p < 0.05; **p < 0.01. The other comparisons were not significant

The proportion of couples with vitrified–thawed–transferred embryos was 47.7%, 46.5%, 61.1%, 33.3% and 47.4% in groups A, B, C, D and E, respectively (Table 4). The five groups did not differ significantly with regard to the pregnancy rates after the transfer of frozen/thawed embryos. However, the CCPR per couple was significantly lower in group D than in the other groups (p < 0.01) (Fig. 2), and the COPR per couple was significantly lower in group D than in group C (p < 0.01) and group E (p < 0.05) (Fig. 2).

**Table 4:** Frozen/thawed embryo transfer outcomes and cumulative clinical and ongoing pregnancy rates/couple

	Group A	Group B	Group C	Group D	Group E	p
N FET cycles	304	260	44	34	182	
N FE transfers	234	236	40	28	168	
Day of FE transfers						
Day 2 or 3 (%)	26.5	23.8	22.5	32.2	22.1	
Day 5 (%)	73.5	76.2	77.5	67.8	77.9	
Frozen embryos per transfer	1.1 ± 0.3	1.1 ± 0.3	1.1 ± 0.2	1.2 ± 0.3	1.1 ± 0.3	
Clinical pregnancy	38	36	8	4	24	
EPL (n)	6	10	0	2	8	
Ectopic pregnancy (n)	0	0	0	0	0	
Twin pregnancy (n)	0	2	0	2	0	
CP/cycle %	12.5	13.8	18.1	11.7	13.1	
CP/transfer %	16.2	15.2	20.0	14.2	14.2	
IR %	14.7	14.6	18.1	17.6	13.0	
OPs	32	26	8	2	16	
OP/cycle %	10.5	10.0	18.1	5.8	8.7	
OP/transfer %	13.6	11.0	20.0	7.1	9.5	
CCPR/couple %	32.5	34.7	38.8	14.8	39.3	**A–D, B–D, C–D, D–E
COPR/couple %	27.2	25.6	33.3	11.1	28.2	*D–E; **C–D

Group A: women without endometriosis; group B: women with endometriosis (all stages); group C: women with stage 1/2 endometriosis; group D: women with stage 3/4 endometriosis (without endometrioma); group E: women with endometrioma alone

*FET* frozen embryo transfer, *EPL* early pregnancy loss, *CP* clinical pregnancy, *OP* ongoing pregnancy, *IR* implantation rate, *CCPR* cumulative clinical pregnancy rate, *COPR* cumulative ongoing pregnancy rate

Statistical significance: *p < 0.05; **p < 0.01. The other comparisons were not significant

## Discussion

Here, we reported pregnancy rates for women with or without endometriosis in our IVF programs. A high proportion (17.9%) of the women attending our ART center (part of the EndoBreizh® regional network) has endometriosis; this high value partly reflects our center’s systematic screening for endometriosis. We found that the pregnancy rates depended on the endometriosis stage (stage 1/2 vs. stage 3/4 vs. endometrioma). Our approach was novel because few studies have distinguished between endometriosis stages [9–13], none has distinguished between endometrioma and other types of stage 3/4 endometriosis, and none has excluded women with adenomyosis. We found that pregnancy rates were similar in women with vs. without endometriosis and similar for stage 1/2 endometriosis vs. endometrioma. However, the clinical and ongoing pregnancy rates were significantly lower for women with stage 3/4 endometriosis (group D), relative to women with endometrioma. These differences were not significant when considering frozen embryo transfers. The CCPR and COPR were significantly lower in group D than other groups. The impact of endometriosis (at any stage) on outcomes in IVF has been extensively studied. For example, Gibbons’ 10-year series of studies at the Jones Institute [16] found similar pregnancy rates among women with endometriosis, women with tubal factors, and women or male causes (respectively 36%, 31% and 32% per retrieval), and the French National Register of In Vitro Fertilization (FIVNAT) [17] found that the cumulative pregnancy rates after 4 cycles were respectively 54.3%, 54.0% and 48.4% in those same groups. Invercini et al. [18] have reported cumulative clinical pregnancy and live birth rates of respectively 50% and 44% in cases of endometriosis vs. 42% and 36% in the absence of endometriosis (p = non-significant). Barnhart et al.’s meta-analysis [7] of IVF programs between 1983 and 1998 found low pregnancy rates for both stage 3/4 endometriosis (13.8% vs. 27.7% in controls; adjusted odds ratio (OR) [95% confidence interval (CI)] 0.46 [0.28–0.74]) and stage 1/2 endometriosis (21.1% vs. 27.7% in controls; 0.56 [0.44–0.70]). Overall, the pregnancy rate was 25.4% in endometriosis and 29.5% for other indications (adjusted OR [95% CI] 0.63 [0.51–0.77]), which contrasts with our present results. Lin et al.’s study [19] of the impact of the woman’s age found a cut-off at 35 y.o: clinical pregnancy rates in IVF were significantly lower in women with endometriosis aged under 35, compared with women without endometriosis in the same age group. In our study, the proportion of women under the age of 35 y.o was similar in groups A and B (53.3%) and similar in groups C, D and E. Hence, we did not observe a cut-off effect.

In our study, we observed significantly lower differences in clinical and ongoing pregnancy rates when comparing women with stage 3/4 endometriosis and those with endometrioma. These results were consistent with Harb et al.’s meta-analysis [11], which found significantly lower implantation rates (risk ratio (RR) [95% CI] 0.79 [0.67–0.93]) and clinical pregnancy rates (RR: 0.79; 95% CI 0.69–0.91) in women with stage 3/4 endometriosis but not in women with stage 1/2 endometriosis. In Rossi and Prefumo’s meta-analysis [9] of 15 studies (encompassing 980 women with endometriosis and 5934 controls), the clinical pregnancy rates were lower in the endometriosis group in general and in women with stage 3/4 endometriosis in particular. There was no difference for the stage 1/2 group, as seen in our study. Similarly, Benaglia et al. [10] found a negative correlation between endometriosis severity and pregnancy rates. Conversely, Maignien et al. [12] found no difference in implantation, clinical pregnancy and birth rates between women with stage 1/2 endometriosis, women with endometrioma, and women with stage 3/4 endometriosis but noted an impact of a lower ovarian reserve and a history of surgery for endometriosis and/or endometrioma. Lastly, Opoien et al. [13] found similar pregnancy rates for stage 1/2, stage 3/4, and tubal etymologies (39.4%, 36.7% and 37.9% per transfer, respectively).

In our study, the significantly lower pregnancy rates in women with stage 3/4 endometriosis (mainly DIE) raise the question of whether these lesions should be removed before admission to an IVF program. Only five women in group D (9.2%) had undergone resection of DIE lesions; in fact, we favor initial IVF unless there is an indication of drug-resistant, severe pain resisting or a risk of urinary or digestive stenosis. Hence, surgery is considered as a second-line treatment when IVF has failed (i.e. more than four good-quality embryos transferred without pregnancy). Our approach is consistent with the publications by Littman et al. [20] and Soriano et al. [21], who operated on women after two IVF failures and observed higher pregnancy rates. Some researchers reported equivalent results in IVF with colorectal endometriosis, such as Capelle et al. [22] and Mathieu d'Argent et al. [23] who reported similar pregnancy rates in women with colorectal endometriosis after the first IVF cycle and in those treated for tubal or male infertility. Conversely, Bendifallah et al. [24] highlighted the value of surgery before IVF, with a pregnancy rate of 49% after surgery vs. 21% in the absence of surgery; p < 0.001), and Ballester et al. [25] noted that pregnancy rates were mostly influenced by adenomyosis. Two meta-analyses by the same group [26, 27] examined pregnancy rates after initial management by surgery or IVF in women with rectovaginal and colorectal lesions (with or without adenomyosis): the clinical pregnancy rates with and without surgery were respectively 37% and 49.8% in the absence of adenomyosis, vs. 11.9% and 40.5% in the presence of adenomyosis. Moreover, adenomyosis was shown to be responsible for a significantly greater early pregnancy loss rate (RR [95% CI] 2.12 [1.2–3.75]) [27]. In our study, we excluded women with adenomyosis and operated on less than 10% of women with stage 3/4 endometriosis before IVF; hence, we cannot draw conclusions about the value of prior removal of these lesions. Stepniewska et al. [28] recommended the complete resection of deep endometriosis lesions but the surgical complication rate ranged between 9 and 23%, with a risk of long-term morbidity (urinary tract and anal problems). Probably in view of these complications, the European Society of Human Reproduction and Embryology [29], the ASRM [6], the Royal College Obstetrics and Gynecology [30] and the Collège national des gynécologues et obstétriciens français [31] consider that surgery is an option after the failure of IVF.

Another question was whether the prior surgical removal of endometriomas was associated with higher pregnancy rates. Endometriomas are present in 20–40% of women with endometriosis [32], although this proportion was much higher in our series (68.7%). Hamdan et al.’s meta-analysis of the impact of endometriomas on IVF results [33] found that pregnancy and birth rates did not depend on the presence of absence of endometriomas but that women with endometriomas had fewer oocytes and more cycle cancellations. After endometrioma surgery, pregnancy and birth rates are similar to those observed for women not having undergone surgery. The pregnancy rates in our study were consistent with the literature data: the numbers of oocytes collected were similar in group E and group A. In our center, we only operate when the endometrioma is more than 60 mm in diameter. We seek to strike a balance between surgery (with better access to ovaries, less contamination of follicular fluids, reduction of infectious risk) and expectant management (no harmful effects on the ovarian reserve). Tsoumpou et al. [34] and Benschop et al.’s Cochrane meta-analysis [35] gave the same results. Conversely, in Opoien et al.’s study [13], the pregnancy and live birth rates were significantly lower in women with endometrioma (26.3% and 18.8%, respectively) than in women without endometrioma (40.2% and 30.5%, respectively).

We mainly used an antagonist GnRH protocol for ovarian stimulation, in accordance with our center’s guidelines. A long agonist GnRH protocol is recommended for cases of associated adenomyosis, which was excluded in our study. A randomized study [36] of the results of antagonist vs. agonist protocols found that there were fewer mature oocytes in the endometrioma group when antagonists were used (with no difference for stage 1/2) but that the implantation and clinical pregnancy rates were equivalent in the two groups. In our study, the fertilization and cleavage rates were similar in all five groups. In Opoien et al.’s study [13], the fertilization rate was significantly lower for stage 1/2 endometriosis, as a result of the lower sperm count in this group. In our study, we observed the same low sperm count in this group but performed significantly more ICSIs, which helped to maintain the fertilization rate. Even though the total gonadotropin dose was significantly higher in group D than in group C, we obtained fewer oocytes, embryos, and especially blastocysts (48.4%, vs. 60% or more in the other groups). The lesser proportion of blastocyst (day 5) transfers might explain the pregnancy rates observed in group D, even if in this group so much pregnancy was obtained after embryo transfer on day 2 or 3 as on day 5. The impact of endometriosis on the embryo has been mentioned by Simon et al. [37] in a study of oocytes donated by women with stage 3/4 endometriosis; after transfer to a woman without endometriosis, the pregnancy rates were abnormally low. Paffoni et al. [38] evidenced the impact of stage 3/4 endometriosis on the embryo, with more blastomere fragmentation and less blastocyst progression (as found in our study). Conversely, Invercini et al. [18] showed that the proportion of good-quality embryos was similar in women with vs. without endometriosis (adjusted OR [95% CI] 0.85 [0.51–1.44]. In Vigano’s study [39], the top-quality cleavage embryo rate was higher in women with endometriosis (vs. controls) but there was no difference in the clinical pregnancy and live birth rates. Endometrial receptivity in endometriosis has also been studied in the context of oocyte donation, with differing results: stage 3/4 endometriosis had no effect on the recipients in Diaz et al.’s study [40] but had a harmful effect on embryo implantation in Papras et al.’s study [41].

## Strengths and limitations

The strengths of our study included the large sample size (with more than 450 cycles in the endometriosis group, although the sizes of the staged groups differed) and the collection of a broad range of demographic and clinical data. Thirdly, we noted whether or not the clinical diagnosis of endometriosis had been confirmed in further examinations. Fourthly, we distinguished between stage 3/4 endometriosis (without endometrioma) and other disease stages. Fifthly, we excluded women with diffuse adenomyosis, as this can bias pregnancy rates in IVF programs. Lastly, we reported the cumulative pregnancy rates. Our study had several limitations, including its retrospective design. There were significant intergroup differences in some important demographic variables but not in the woman’s age, BMI, smoking status, primary cause of infertility, and normal uterine status (i.e., predictors of pregnancy).

## Conclusions

Our study showed that pregnancy rates were similar in IVF in women with endometriosis and women without endometriosis but that women with stage 3/4 endometriosis had significantly lower cumulative pregnancy rates. These results are consistent with some of the literature data [42]. The value of initial surgical management of DIE before IVF remains subject to debate [43]. Other treatments for endometriosis (e.g., aromatase inhibitors and dienogest) [44] might improve IVF outcomes. More prospective studies are needed to validate the IVF results and determine which women with endometriosis have the best IVF prognosis [45].

## Data Availability

The material contained in this manuscript has not been published, has not been submitted or is not being submitted elsewhere. The datasets used and/or analysed during the current study are available from the corresponding author on reasonable request.

## Abbreviations

AFS: American Fertility Society
AMH: Anti-Müllerian hormone
ART: Assisted reproductive technology
ASRM: American Society of Reproductive Medicine
BMI: Body mass index
CCPR: Cumulative clinical pregnancy rate
COPR: Cumulative ongoing pregnancy rate
CI: Confidence interval
dhCG: Day of hCG
DIE: Deep infiltrating endometriosis
d: Day
E2: Estradiol-17β
EPL: Early pregnancy loss
FE: Frozen embryo
FET: Frozen (thawed) embryo transfer
FSH: Follicle stimulating hormone
GnRH: Gonadotropin releasing hormone
hCG: Human chorionic gonadotropin
hMG: Human menopausal gonadotropin
ICSI: Intracytoplasmic sperm injection
IU: International unit
IVF: In vitro fertilization
LH: Luteinizing hormone
MII: Mature oocyte in metaphase II
opu: Oocyte pick-up
OR: Odds ratio
PCOS: Polycystic ovary syndrome
y.o: Years old
